# Ibutilide treatment protects against ER stress induced apoptosis by regulating calumenin expression in tunicamycin treated cardiomyocytes

**DOI:** 10.1371/journal.pone.0173469

**Published:** 2017-04-11

**Authors:** Yu Wang, Liying Xuan, Xiaoxue Cui, Yilin Wang, Shaoqing Chen, Chengxi Wei, Ming Zhao

**Affiliations:** 1 Medicinal Chemistry and Pharmacology Institute, Inner Mongolia University for the Nationalities, Tongliao, Inner Mongolia, P.R. China; 2 Inner Mongolia Provincial Key Laboratory of Mongolian Medicine Pharmacology for Cardio-Cerebral Vascular System, Tongliao, Inner Mongolia, P.R. China; 3 First Clinical Medical College of Inner Mongolia University for Nationalities, Tongliao, Inner Mongolia, P.R. China; Duke University School of Medicine, UNITED STATES

## Abstract

**Background:**

Ibutilide, a class III antiarrhythmic agent has been shown to be cardioprotective in treating atrial fibrillation, promoting cardioconversion and recently this agent has been shown to protect against ER stress induced apoptosis in cardiomyocytes. In this study we begin to identify the mechanism by which ibutilide exerts its cardioprotection in tunicamycin treated cardiomyocytes. We examined ER stress markers including calumenin; a calcium binding ER chaperone protein that has recently been linked to ER stress in cardiomyocytes, in our treated cells.

**Methods:**

To assess the effect of ibutilide we used the well characterized in vitro model of ER stress induced apoptosis in rat neonatal cardiomyocytes (RNC). RNC were treated with tunicamycin and the degree of ER stress was assessed by quantifying mRNA and protein levels of GRP78, GRP94 and calumenin, and examined the extent of apoptosis by assessing the protein levels of caspase-3/9/12, CHOP, ATF6, p-PERK, spliced XBP-1, the ratio of Bax/Bcl-2 and the percentage of deoxynucleotidyl-transferase- mediated dUTP nick end labeling (TUNEL) positive cells.

**Results:**

We demonstrate ibutilide attenuated the up-regulation of ER stress markers GRP78 and GRP94 and rescued the decline in calumenin mRNA and protein levels in tunicamycin treated cardiomyocytes. The up-regulation of apoptotic markers caspase-3, CHOP, ATF6, p-PERK, spliced XBP-1, the ratio of Bax/Bcl-2 and the percentage of TUNEL positive cells were also attenuated after ibutilide treatment while the protein levels of Caspase-9 and Caspase-12 were unaffected.

**Conclusions:**

This study suggests another cardioprotective effect of the antiarrhythmic agent ibutilide whereby pretreatment leads to the attenuation of ER stress induced apoptosis by regulating calumenin expression. This study provides further evidence for the role of calumenin in the cardiomyocyte ER stress response.

## Introduction

Myocardial apoptosis has been shown to contribute to the pathogenesis of various heart diseases promoting structural remodeling and alterations in heart function [1]. Specifically, intrinsic apoptosis mediated by mitochondrial proteins have been shown to lead to the pathogenesis of myocardial infarction and heart failure [2]. Support for this mitochondrial centered cell death has also been confirmed in atrial fibrillation (AF) patients. Examination of specimens derived from the myocardium of these patients revealed enhanced expression of the pro-apoptotic protein Caspase-3, lowered expression of the anti-apoptotic protein BCL-2 and enhanced deoxynucleotidyl-transferase- mediated dUTP nick end labeling (TUNEL) positive cells were observed in AF patient samples as compared to non-diseased samples. A decreased left ventricular ejection fraction was observed to correspond to this histological profile [3, 4]. Due to the irreversible nature of this myocardial remodeling treatments that prevent or terminate the activation of this apoptotic pathway are necessary.

We are just beginning to understand the pathways that initiate myocardial apoptosis in disease. Recently, endoplasmic reticulum (ER) stress induced apoptosis has become a focus. In the heart, the ER regulates Ca^2+^ homeostasis, excitation contraction coupling and is the site of folding, synthesis and structural maturation of more than a third of all proteins made in the cell [5]. Dysregulation of these processes, as observed in the heart during pressure overload and ischemia/ reperfusion have been shown to lead to ER stress and activation of the unfolded protein response (UPR) [2]. In mouse hearts exposed to ischemia reperfusion UPR is initiated as indicated by the up-regulation of ER resident chaperone proteins GRP78 and GRP94 [6]. These chaperone proteins attempt to re-establish ER homeostasis by sensing improperly folded proteins and promoting Ca^2+^ buffering [7, 8] however under conditions of prolonged ER stress apoptotic pathways are activated. Recently, Lee Jh et al found the calcium binding ER chaperone protein calumenin plays a role in ER stress induced apoptosis whereby over-expression of calumenin reduced ER stress and apoptosis in tunicamycin treated cardiomyocytes. This study highlights the importance of calumenin protein levels in maintaining cardiomyocyte homeostasis [9]. Identifying agents that attenuate ER stress and ultimately prevent cardiomyocyte apoptosis is essential to prevent heart failure in diseased cardiomyocytes.

Dysregulation of Ca^+2^ homeostasis in cardiomyocytes is directly linked to ER stress and apoptosis in ischemia/ reperfusion injury [10] and recently dysregulation of membrane hyperpolarization by the inward rectifier K^+^ channel has been linked to alterations in intracellular Ca^2+^ concentration and cell death in the immortalized bovine brain endothelial cell line t-BBEC117 [11]. Agents that regulate cardiomyocyte membrane potential may prove to have beneficial effects in treating ER stress induced apoptosis. Ibutilide is class III cardioconversion agent commonly used to treat recent onset atrial fibrillation (AF) patients. Intravenous administration of this drug lengthens the repolarization period, action potential, and refractory period of atrial and ventricular myocardium by blocking the delayed rectifier potassium current and acutely extending the inward slow Na^+^ current [12].

In this study using an in vitro system we were able to initiate ER stress mediated apoptosis in rat neonatal cardiomyocytes by the application of tunicamycin. This ER stress inducing agent promoted the enhanced mRNA and protein expression of ER stress markers GRP78 and GRP94 and lowered expression levels of calumenin, providing further evidence of the importance of maintaining calumenin expression levels for cardiomyocyte homeostasis. These findings highlight the potential diagnostic and therapeutic potential of regulating ER chaperone proteins such as calumenin in treating heart disease.

Tunicamycin treatment also increased protein levels of the pro-apoptotic proteins CHOP, ATF6, Caspase-3, Caspase-9 and Caspase-12 and the Bax/ Bcl-2 ratio was increased, with a corresponding increase in TUNEL positive cells. For the first time we identified pre-treatment of tunicamycin treated cardiomyocytes with ibutilide attenuated the extent of up-regulation of mRNA and protein levels of ER stress markers and preserved the expression level of calumenin. The extent of up- regulation of protein levels of the pro-apoptotic proteins were also attenuated with ibutilide pre-treatment with a corresponding decline in the percentage of TUNEL positive cells. We also saw when silencing endogenous expression of calumenin the protective effect of ibutilide was lost. This study introduces a previously uncharacterized effect of ibutilide in preventing ER stress induced apoptosis in cardiomyocytes by the regulation of calumenin expression. This study also highlights the versatility of this agent and potential for use beyond treating AF patients.

## Material and methods

### Ethics statement

Rats related with this research was in accordance with the NIH Guide for the Care and Use of Laboratory animals and all protocols in this study were approved by the Institutional Animal Care and Use Committee of Inner Mongolia University for the Nationalities (NM-Y-2015-03-13-14-001)

### Animals

1–3 day neonatal rats were purchased from Inner Mongolia Nationalities University Animal Center. They were housed in a room with a constant airflow system, a 12 hour light/dark cycle and controlled temperature (21–23°C). Neonatal rats were sacrificed by decapitation.

### Rat neonatal cardiomyocytes isolation and culture

1–3 day neonatal rats were euthanized by decapitation, hearts excised and ventricular were removed. Atria were isolated by type II collagenase as previously described [13]. At the end of each cycle, the suspension was centrifuged, the supernatant collected and kept on 4°C. The supernatants were pooled, centrifuged and cultured in standard DMEM:F12 supplemented with 10% FBS and 1% penicillin and streptomycin.

### In vitro model of ER stress induced apoptosis

In order to model ER stress induced apoptosis we treated RNC with 5μg/ml tunicamycin for 30 minutes. Cells in the *treatment group* were first pretreated with 10 μg/ml of ibutilide for 30minutes before being exposed to 5μg/ml of tunicamycin for 30 minutes.

### Cell transfections

Neonatal rat cardiomyocytes were cultured on cell culture grade plates for 24 hour and transfected with either control or calumenin siRNA. siRNA oligonucleotides targeting calumenin mRNA in rat neonatal cardiomyocytes were designed (sequence: 5’ ggatggagacctaattgcc 3’) and then inserted into the pGCSIL-GFP vector and transfected into cells to knock down calumenin protein expression. Lipo2000 was used to transfect cells. Media was changed every 24 h. Cells were analyzed under an AX70 epifluorencent microscope (Olympus) to examine transfection efficiency. The experiments were conducted 72 hr after transfection.

### Reagents

Super M-MLV reverse transcriptase was purchased from BioTeke. RNA simple total RNA Kit was acquired from TIANGEN (Beijing, China). Terminal deoxynucleotidyl transferase-mediated dUTP nick end-labeling (TUNEL) assay kit was purchased from Beyotime Biotechnology (ShangHai, China). Ibutilide and tunicamycin were obtained from SIGMA (USA).

### TUNEL assay

Following the manufacturer’s instructions, DNA fragmentation of apoptotic cells was detected by TUNEL staining and the number of apoptotic cells, as defined by chromatin condensation of nuclear fragmentation (apoptotic bodies) was counted.

### Real-time PCR

The RNA of cardiomyocytes was isolated using an RNA extraction kit (TIANGEN), and then isolated RNA was transcribed. The obtained cDNA was used for real-time PCR.

The primers of calumenin, CX40, CX43, GRP78, GRP94, GAPDH gene fragments were designed following:

Calumenin-F: 5’ ACACTTTCTCAATCCCTTACC 3’

Calumenin-R: 5’ CTGGGCCTGTGACAACTG 3’

GRP78-F: 5’ GATAATCAGCCCACCGTAA 3’

GRP78-R: 5’ TTGTTTCCTGTCCCTTTGT 3’

GRP94-F: 5’ GATGTGGATGGCACGGTAG3’

GRP94-R: 5’ GTTCCCTTATTTGTGATGCA 3’

GAPDH F: 5’ CGGCAAGTTCAACGGCACAG 3’

GAPDH R: 5’ CGCCAGTAGACTCCACGACAT 3’

Amplification was performed in duplicate on FTC-3000 Real-Time PCR system thermocycler using SYBR Green PCR Master Mix (TIANGEN, Beijing, China). The relative mRNA expression level of the gene was normalized to the level of GAPDH in the same sample.

### Western blot analysis

Western blot was performed as described previously [14]. Antibodies for anti-calumenin (1: 500), anti-caspase-3 (1: 500), anti-caspase-9 (1: 500), anti-caspase-12 (1: 500) were obtained from Bioss, anti-CHOP (1: 1000), anti-Bcl-2 (1: 1000) were obtained from Wanleibio, and anti-GRP78 (1: 400), anti-GRP94 (1: 400), anti-Bax (1: 400), anti-ATF-6 (1: 400) were purchased from Boster, and anti-p-PERK (1:750), anti-PERK (1:750), anti-spliced XBP-1 (1:500), anti-unspliced XBP-1 (1:500) were obtained from Abcam. Protein was extracted and mixed in loading buffer, and then equal amounts were fractionated on gel and transferred onto Hybond-C Extra nitrocellulose membrane using a semidry transfer apparatus. Protein was blocked with nonfat dry milk and detected with supersignal west pico chemiluminescent substrate.

### Statistical analysis

All values were expressed as mean ± SE. Multi-group comparisons of the means were carried out by matched t-test using SPSS 11.5.

## Results

### Ibutilide attenuates tunicamycin-induced apoptosis

To induce ER stress mediated apoptosis we used the well characterized natural compound tunicamycin. This antibiotic has been shown to mediate apoptosis by inhibiting glycosylation and proper folding of proteins and promoting ER stress [13] and recently this compound has specifically been shown to induce ER stress mediated apoptosis in rat neonatal cardiomyocytes (RNC) [14].

**Fig 1:**
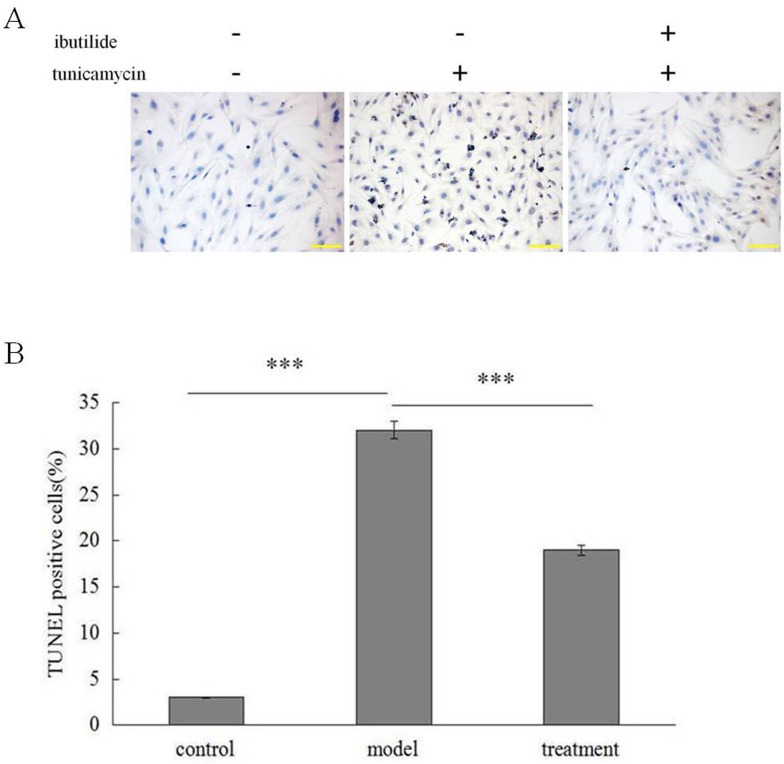
Ibutilide’s protective effect on tunicamycin induced apoptosis in RNC. TUNEL staining was quantified in cells treated with tunicamycin alone (model), tunicamycin pre-treated with ibutilide (treatment) or untreated RNC (control). (A) Representative bright field images. (B) Quantification of TUNEL positive cells. All data are shown as mean ± SE (*n* = 3 per group). ***p<0.001.

To examine the extent of ER stress induced apoptosis in our system we performed terminal deoxynucleotidyl-transferase- mediated dUTP nick end labeling (TUNEL) on RNC treated with tunicamycin. Treatment lead to a 10 fold increase in the percentage of TUNEL positive cells as compared to non-treated cells. Pre-treatment of RNC with the cardioconversion drug ibutilide decreased the percentage of TUNEL positive cells by half (Fig 1A and 1B). These findings confirm tunicamycin treatment induces apoptosis in RNC and pre-treatment with ibutilide attenuates the extent of apoptosis.

### Ibutilide attenuates the extent of tunicamycin induced activation of the intrinsic apoptotic pathway

**Fig 2:**
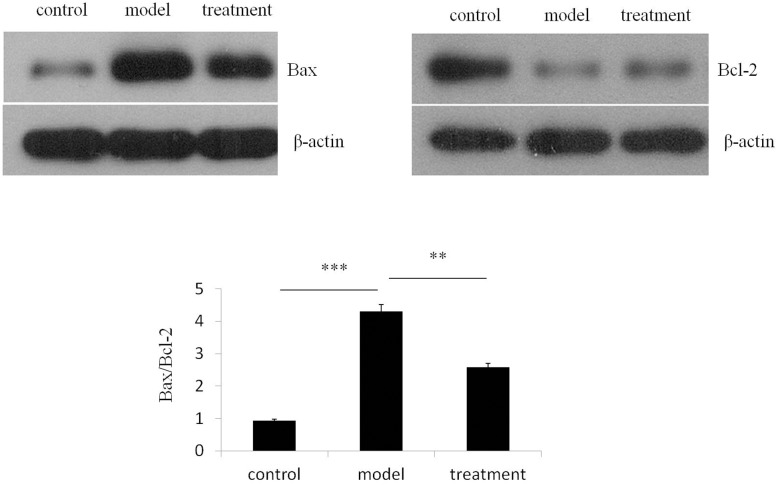
Ibutilide treatment attenuates tunicamycin induced increase in Bax/ Bcl-2 ratio. Protein expression of Bax and Bcl-2 were analyzed from lysate derived from RNC treated with either tunicamycin alone (model), tunicamycin and ibutilide (treatment) or untreated cardiomyocytes (control). Representative immunoblots of lysate immunoblotted with antibodies specific for Bax or BCL-2 are shown. The quantification of normalized band densitometry of Bax was divided by that of Bcl-2 and the results are graphed. All data are shown as mean ± SE (*n* = 3 per group). *p<0.5, **p<0.01, ***p<0.001.

**Fig 3:**
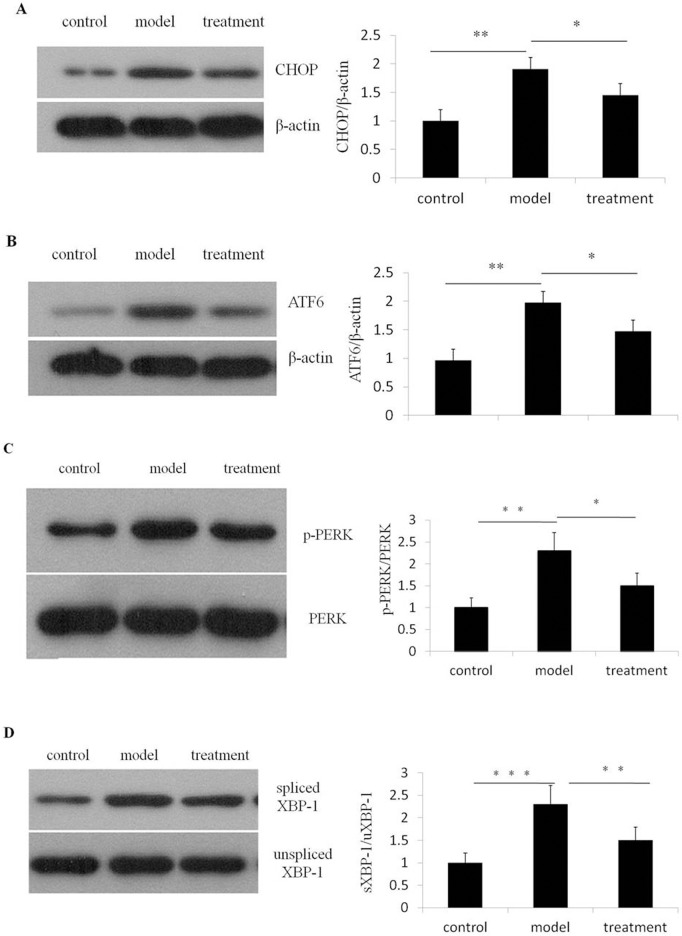
Ibutilide treatment attenuates tunicamycin induced up-regulation of CHOP, ATF6, p-PERK and spliced XBP-1 protein expression. Protein expression of CHOP, ATF6, p-PERK and spliced XBP-1 were analyzed from lysate derived from RNC treated with either tunicamycin alone (model), tunicamycin and ibutilide (treatment) or untreated cardiomyocytes (control). Representative immunoblots of lysate immunoblotted with antibodies specific for CHOP (A), ATF6 (B), p-PERK (C) and spliced XBP-1 (D) are shown. Lysate were immunoblotted for β-actin as a loading control. The quantification of normalized band densitometry is graphed adjacent to its corresponding immunoblot. All data are shown as mean ± SE (*n* = 3 per group). *p<0.5, **p<0.01, ***p<0.001.

To further examine the protective effect of ibutilide on apoptosis we examined protein levels of the pro-apoptotic markers Bax, CHOP, ATF6, PERK, p-PERK, spliced XBP-1 and unspliced XBP-1, Caspase-3, Caspase -9 and Caspase-12 after tunicamycin treatment. Tunicamycin treatment of rat neonatal ventricular cardiomyocytes has previously been shown to increase expression levels of several members of the intrinsic pro-apoptotic pathway including CHOP [9]. In our study tunicamycin treatment lead to a four- fold up-regulation of the Bax/Bcl2 ratio in RNC while ibutilide pre-treatment attenuated this up-regulation by half, due to the up-regulation of Bcl2 expression observed after treatment with this agent (Fig 2). The tunicamycin induced up-regulation of ERS markers, including phosphorylated PERK (p-PERK), spliced XBP1, ATF6 and CHOP (Fig 3) were also attenuated after ibutilide treatment.

**Fig 4:**
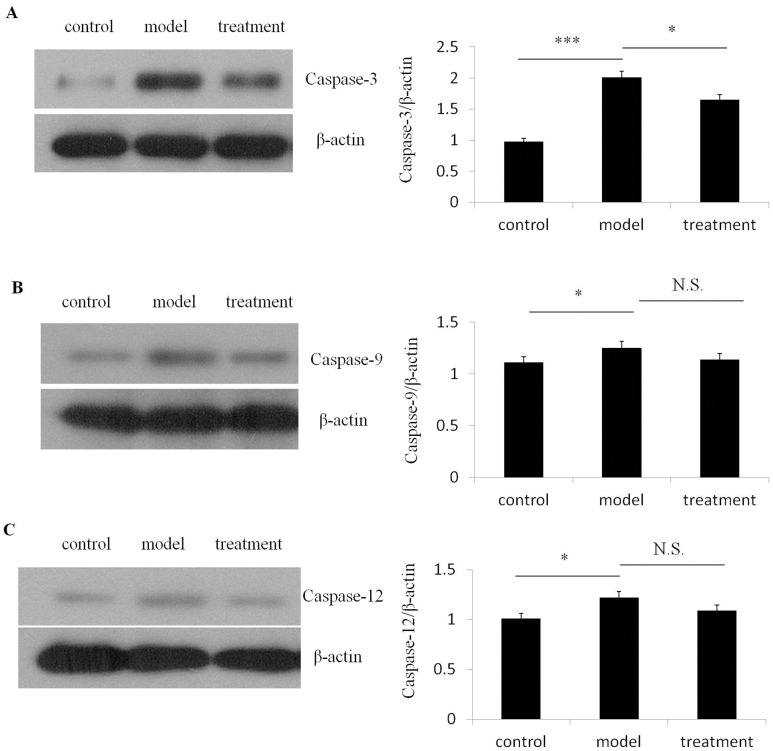
Ibutilide treatment attenuates tunicamycin induced up-regulation of Caspase-3 protein expression. Protein expression of Caspase-3, Caspase-9 and Caspase-12 were analyzed from lysate derived from RNC treated with either tunicamycin alone (model), tunicamycin and ibutilide (treatment) or untreated cardiomyocytes (control). Representative immunoblots of lysate immunoblotted with antibodies specific Caspase-3 (A), Caspase-9 (B) or Caspase-12 (C). Lysates were immunoblotted for β- actin as a loading control. The quantification of normalized band densitometry is graphed adjacent to its corresponding immunoblot. All data are shown as mean ± SE (*n* = 3 per group). *p<0.5.

Finally, we analyzed the expression of Caspases-3/9/12. We observed ibutilide treatment attenuated the extent of tunicamycin induced up-regulation of Caspase-3 however the level of Caspase-9 and Caspase-12 were not significantly affected by ibutilide (Fig 4). These findings for the first time identify ibutilide attenuates the extent of tunicamycin induced activation of the intrinsic apoptotic pathway in cardiomyocytes.

### Tunicamycin induced ER stress is attenuated in Ibutilide pre-treated RNC

**Fig 5:**
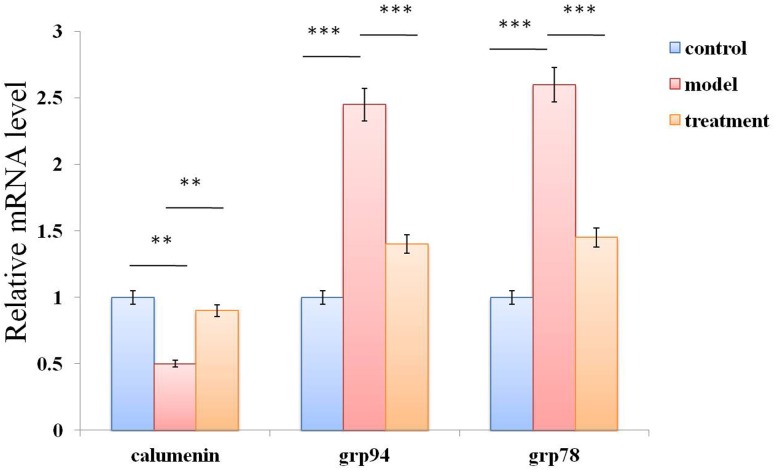
Ibutilide treatment alters mRNA levels of ER stress markers GRP78, GRP94 and calumenin in tunicamycin treated RNC. mRNA expression of GRP78, GRP94 and calumenin were analyzed by real-time PCRin RNC treated with either tunicamycin alone (model), tunicamycin pre-treated with ibutilide (treatment) or untreated cells (control). All data are shown as mean ± SE (*n* = 3 per group). **p<0.01, ***p<0.001.

ER stress has been shown to activate the intrinsic apoptotic pathway in cardiomyocytes and has been suggested to play a crucial role in the pathogenesis of heart disease [14, 15]. We examined the effects of tunicamycin treatment on mRNA levels of ER stress markers GRP78 and GRP94 in RNC using RT-PCR. We observed exposure of these cells to the ER stress inducing agent promoted up-regulation of these ER stress markers. Recently the ER chaperone calumenin has been shown to regulate ER stress induced apoptosis in cardiomyocytes and decreased levels of expression have been shown to promote ER stress and apoptosis [9]. We examined mRNA levels of the cardioprotective ER chaperone calumenin in tunicamycin treated cells and observed decreased levels of expression. Ibutilide pre-treatment of tunicamycin exposed cells prevented the decline in calumenin and suppressed the upregulation of GRP78 and GRP94 (Fig 5).

**Fig 6:**
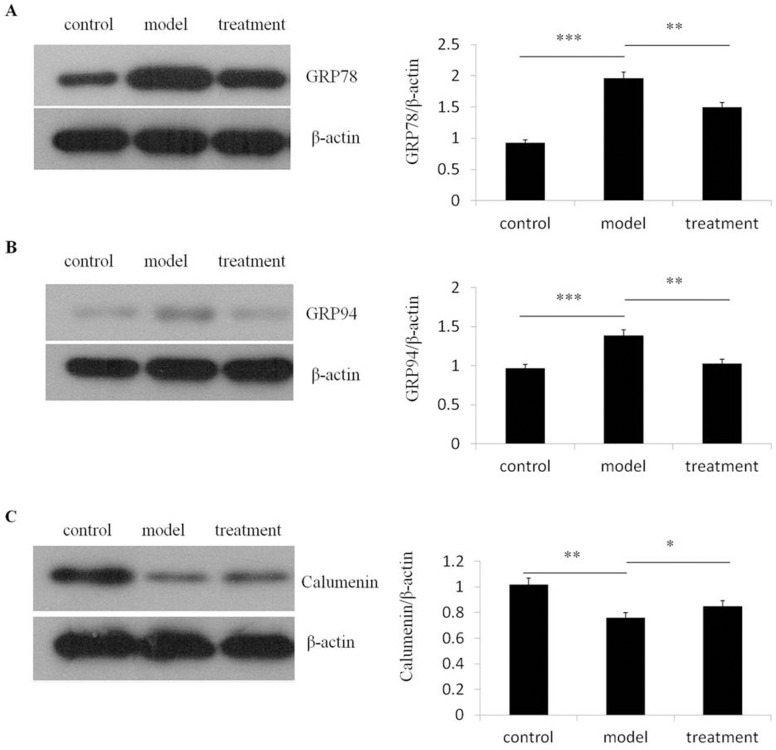
Ibutilide treatment alters protein expression of ER stress markers GRP78, GRP94 and calumenin in tunicamycin treated RNC. Protein levels GRP78 and GRP94 and calumenin were analyzed from lysates derived from RNC treated with either tunicamycin alone (model), tunicamycin and ibutilide (treatment) or untreated cardiomyocytes (control). Representative immunoblots of lysate immunoblotted with antibodies specific for (A) GRP78, (B) GRP94 or (C) Calumenin. Quantification of band densitometry is shown adjacent to the corresponding blot. All data are shown as mean ± SE (*n* = 3 per group). *p<0.5, **p<0.01, ***p<0.001.

Protein expression of these ER stress markers were also examined by western blotting lysate derived from these treated cells (Fig 6). Protein levels of GRP78 and GRP94 were enhanced 2 fold by tunicamycin treatment (model) and calumenin levels declined. Pre-treatment of cells with ibutilide attenuated the degree of upregulation of GRP78 and completely prevented the upregulation of GRP94. The degree of tunicamycin induced downregulation of calumenin was attenuated by ibutilide pre-treatment although to a lesser extent then the rescue observed at the mRNA level, suggesting the protective effect of ibutilide may be at the transcriptional level.

**Fig 7:**
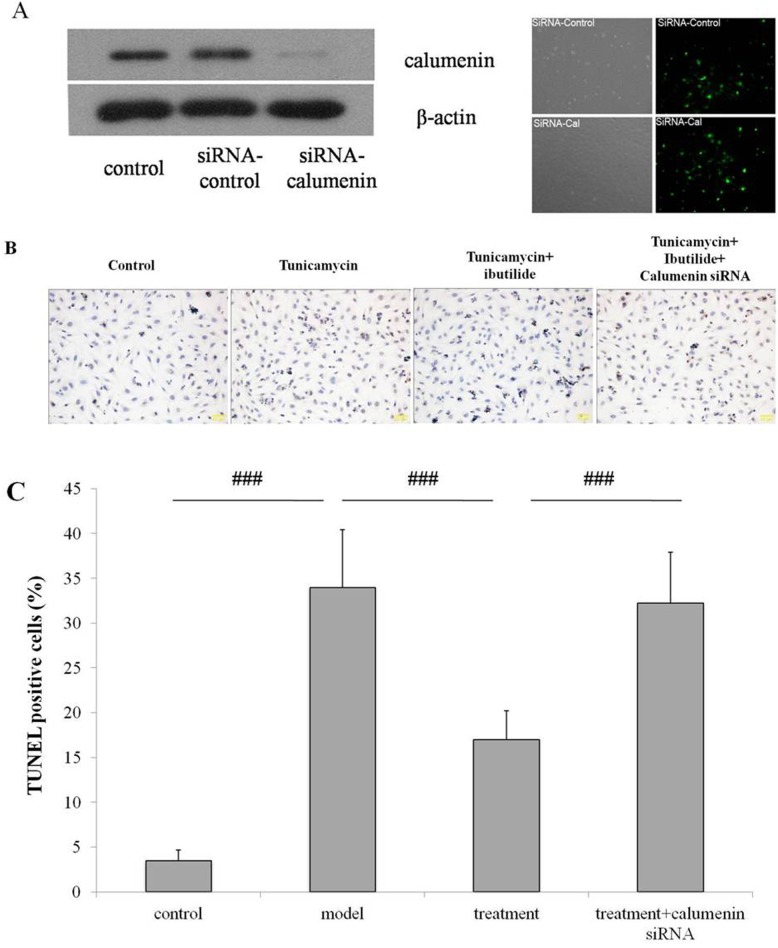
Ibutilide attenuates tunicamycin induced apoptosis in RNC through calumenin. (A) Calumenin protein expression was determined in non- transfected cells (control), cells transfected with control siRNA and calumenin specific siRNA (left panel); transfection efficiency was examined in cells transfected with siRNA-control and siRNA-Calumenin using fluorescent microscopy. (B) TUNEL staining was quantified in non-treated cells (control), RNC treated with tunicamycin (model), RNC treated with ibutilide and tunicamycin (treatment), and RNC transfected with calumenin siRNA and treated with tunicamycin and ibutilide. (C) Representative bright field images at 200x magnification. Quantification of TUNEL positive cells. All data are shown as mean ± SE (*n* = 3 per group). ***p<0.001

To further examine if ibutilide modulates apoptosis via calumenin, we down-regulated calumenin expression by RNA interference in RNC. The expression of calumenin was examined by western blotting. We confirmed attenuation of calumenin protein levels after transfection with calumenin specific siRNA (Fig 7A left panel). The GFP fluorescent signals were imaged to verify the successful transfection (Fig 7A right panel). Next we used the TUNEL assay to examine the extent of apoptosis in RNC where calumenin expression was attenuated. Silencing calumenin expression in ibutilide and tunicamycin treated cells lead to a 2 fold upregulation of TUNEL positive cells. (Fig 7B and 7C). These findings confirm ibutilide attenuates tunicamycin treatment induces apoptosis in RNC through caulumenin.

## Discussion

This study for the first time identifies ibutilide has the ability to attenuate ER stress induced apoptosis in cardiomyocytes by regulating calumenin expression. To identify this previously unknown mechanism of ibutilide mediated cardioprotection we treated rat neonatal cardiomyocytes (RNC) with the well characterized ER stress inducing agent tunicamycin. Tunicamycin has previously been shown to initiate ER stress induced apoptosis in various cells types by preventing glycosylation and protein folding and inducing prolonged ER stress in treated cells [13]. We observed pre-treatment of RNC with ibutilide attenuated the extent of tunicamycin induced ER stress, activation of the apoptotic pathway and ultimately the percentage of TUNEL positive cardiomyocytes.

Ibutilide a class III antiarrhythmic drug is acutely administered to recent onset atrial fibrillation (AF) patients to promote cardioconversion. This agent enhances the refractory period in the action potential of AF patients by activating slow sodium ion channels [12] and deactivating delayed rectifier potassium channels [16]. Recently, tunicamycin treatment was shown to up- regulate mRNA and protein levels of the inward rectifying K^+^ channel 2.1 (Kir 2.1) in brain capillary endothelial cells with a corresponding increase in inward rectifier K+ current. This increased current lead to the establishment of a deeply hyperpolarized resting membrane potential, increasing the intracellular Ca^2+^ concentration due to enhanced Ca^2+^ entry from non-selective cation channels and promoting cell death/ cell turnover of treated cells [17].

Tunicamycin has also been shown to promote altered calcium and ROS signaling in treated cardiomyocytes which led to ion channel dysregulation, contractile dysfunction and ultimately cell death [18]. Based on these observations it is reasonable to hypothesize that one of the ways that tunicamycin initiated ER stress induced apoptosis in our treated RNC is by altering the expression levels of membrane ion channels and/or activity of these ion channels which leads to alterations in cellular membrane potential, most likely hyperpolarization. This state of membrane hyperpolarization may have lead ultimately to dysregulation of intracellular calcium dynamics in our treated RNC. An examination of calcium dynamics in our tunicamycin treated RNC cells are necessary to verify this hypothesis along with a characterization of altered ion channel expression and activity including assessment of K^+^ channel family members to verify that tunicamycin promotes Ca^2+^ dysregulation in cardiomyocytes.

In our study we observed ibutilide pre-treatment attenuated the degree of up-regulation of the ER stress markers GRP78 and GRP94 at both the mRNA and protein level. We also observed the degree of downregulation of the newly characterized ER stress marker calumenin, was also attenuated after ibutilide treatment. The maintenance of expression levels of calumenin has been previously shown to be cardioprotective against tunicamycin initiated ER stress induced apoptosis in treated cardiomyocytes [9]. In our study we confirmed this finding and showed one of the mechanisms by which ibutilide exerts its cardioprotective role is by maintaining expression levels of calumenin. Specifically, when we silenced calumenin expression and treated cells with tunicamycin, the protective effect of ibutilide was lost.

Based on the known pharmacokinetics of ibutilide as mentioned above, we propose the observed attenuation of ER stress and prevention of altered expression levels of these ER chaperone proteins by tunicamycin is by preventing the extent of tunicamycin induced hyperpolarization of the membrane potential, maintaining physiological Ca^2+^ and ROS levels and inhibiting activation of UPR, ER stress and the apoptotic signaling pathway. Recent studies have confirmed the protective role of ibutilide against oxidation stress in cardiomyocytes [19] but further measurements must be taken of other UPR proteins to determine what arm or arms of the UPR pathway is regulated by ibutilide in tunicamycin treated cells.

The role of the mitochondrial mediated intrinsic apoptotic pathway in mediating ER stress induced apoptosis is well characterized in cardiomyocytes [9, 14]. In our study we observed the extent of tunicamycin induced activation of this pathway and the percentage of TUNEL positive RNC was also attenuated by ibutilide pre-treatment specifically, the degree of tunicamycin initiated up-regulation of Bax, CHOP, ATF-6, p-PERK, spliced XBP-1 and Caspase-3 was attenuated by ibutilide. We also observed that BCL-2 expression was up-regulated in ibutilide treated RNC as compared to cells treated with tunicamycin, a finding that would suggest that this agent may promote the activation of the anti-apoptotic pathway at the transcriptional level in response to tunicamycin treatment. Although we have not experimentally determined the mechanism by which ibutilide attenuates apoptosis we can hypothesize that this agent may prevent ER stress activated transcription factors, like CHOP [20] from initiating a pro- apoptotic program.

Interestingly we observed ibutilide pre-treatment effected the level of caspase-3 but did not affect the expression levels of initiator caspases -9 and caspase-12. These differences may be due to the versatility of caspase-3 in mediating apoptosis as this protein has been shown to be involved in both the intrinsic [21] and extrinsic apoptotic pathway [22] in cardiomyocytes. Although the immune roles of Caspase-12 are still controversial, its expression is regulated by inflammation and it play negative roles in pro-inflammatory responses by inactivating caspase-1 and other inflammatory pathways. Our observation that ibutilide did not affect caspase-12 expression level suggests that ibutilide may not have protective roles in cellular inflammation in cardiomyocytes. As caspase-9 is related to mitochondria-mediated apoptosis, minor effect of ibutilide on caspase-9 expression suggests that ibutilide alleviates ER stress and apoptosis bypassing mitochondrial pathways. These findings suggest that ibutilide’s attenuation of UPR and ER stress corresponds to a decline in the extent of activation of apoptosis by regulating the expression levels of the ER chaperone protein calumenin. This study also highlights its potential therapeutic use in treating heart diseases where myocardial apoptosis is present.
